# The evidence base of primary research in public health emergency preparedness: a scoping review and stakeholder consultation

**DOI:** 10.1186/s12889-015-1750-1

**Published:** 2015-04-28

**Authors:** Yasmin Khan, Ghazal Fazli, Bonnie Henry, Eileen de Villa, Charoula Tsamis, Moira Grant, Brian Schwartz

**Affiliations:** Public Health Ontario, 480 University Avenue, Suite 300, Toronto, ON M5G 1V2 Canada; Division of Emergency Medicine, Department of Medicine, University of Toronto, 2075 Bayview Ave, C753, Toronto, ON M4N 3M5 Canada; School of Public Health and Health Systems, University of Waterloo, 200 University Avenue West, Waterloo, ON N2L 3G1 Canada; British Columbia Centre for Disease Control, 655 W 12th Ave, Vancouver, BC V5Z 4R4 Canada; School of Population and Public Health, Faculty of Medicine, University of British Columbia, 2206 East Mall, Vancouver, BC V6T 1Z3 Canada; Peel Public Health, 7120 Hurontario Street, P.O Box 667 - RPO Streetsville, Mississauga, ON L5M 2C2 Canada; Dalla Lana School of Public Health, University of Toronto, 155 College St, Toronto, ON M5T 3M7 Canada

## Abstract

**Background:**

Effective public health emergency preparedness and response systems are important in mitigating the impact of all-hazards emergencies on population health. The evidence base for public health emergency preparedness (PHEP) is weak, however, and previous reviews have noted a substantial proportion of anecdotal event reports. To investigate the body of research excluding the anecdotal reports and better understand primary and analytical research for PHEP, a scoping review was conducted with two objectives: first, to develop a thematic map focused on primary research; and second, to use this map to inform and guide an understanding of knowledge gaps relevant to research and practice in PHEP.

**Methods:**

A scoping review was conducted based on established methodology. Multiple databases of indexed and grey literature were searched based on concepts of public health, emergency, emergency management/preparedness and evaluation/evidence. Inclusion and exclusion criteria were applied iteratively. Primary research studies that were evidence-based or evaluative in nature were included in the final group of selected studies. Thematic analysis was conducted for this group. Stakeholder consultation was undertaken for the purpose of validating themes and identifying knowledge gaps. To accomplish this, a purposive sample of researchers and practicing professionals in PHEP or closely related fields was asked to complete an online survey and participate in an in-person meeting. Final themes and knowledge gaps were synthesized after stakeholder consultation.

**Results:**

Database searching yielded 3015 citations and article selection resulted in a final group of 58 articles. A list of ten themes from this group of articles was disseminated to stakeholders with the survey questions. Survey findings resulted in four cross-cutting themes and twelve stand-alone themes. Several key knowledge gaps were identified in the following themes: attitudes and beliefs; collaboration and system integration; communication; quality improvement and performance standards; and resilience. Resilience emerged as both a gap and a cross-cutting theme. Additional cross-cutting themes included equity, gender considerations, and high risk or at-risk populations.

**Conclusions:**

In this scoping review of the literature enhanced by stakeholder consultation, key themes and knowledge gaps in the PHEP evidence base were identified which can be used to inform future practice-oriented research in PHEP.

**Electronic supplementary material:**

The online version of this article (doi:10.1186/s12889-015-1750-1) contains supplementary material, which is available to authorized users.

## Background

For more than a decade, there has been substantial attention to and investment in emergency preparedness and response capacity for emergencies with health impacts. The importance of robust emergency preparedness and response systems for health emergencies is highlighted by recent incidents such as the Ebola outbreak in West Africa, the emergence of Middle East Respiratory Syndrome Coronavirus, natural disasters such as floods, and industrial-technical incidents like train derailments [1-4]. Preparedness for the diversity of events that may impact health is described as an ‘all-hazards’ approach, highlighting the importance of ensuring the system is prepared for a variety of potential threats. While there are multiple sectors with responsibilities for emergency preparedness and response, actions taken to prepare and respond to the population health consequences of emergencies fall to public health emergency preparedness (PHEP). PHEP has been defined as “the capability of the public health and health care systems, communities, and individuals, to prevent, protect against, quickly respond to, and recover from health emergencies, particularly those whose scale, timing, or unpredictability threatens to overwhelm routine capabilities” [5]. Examples of actions of PHEP related to health emergencies include surveillance and epidemiologic activities to monitor, detect, and investigate potential health threats, and the development and communication of information to the public [5]. In the context of the Severe Acute Respiratory Syndrome (SARS) outbreak of 2003, gaps were noted in the Canadian public health system including coordinated surveillance systems for detecting outbreaks and communication to the public [6].

Despite the investments into operational capacity for PHEP, a persistent challenge is a paucity of evidence to inform and guide practice [7-11]. Evidence-based approaches to public health practice have been recognized as crucial [12], alongside established practice in related fields of evidence-based medicine and health decision-making [13-16]. Evidence-based or informed practice in public health encompasses incorporating scientific evidence in selecting and implementing programs, developing policies, and evaluating progress [12]. In the PHEP field, however, anecdotal responses to recent events are typically used as the best available science [10]. A review of the emergency planning literature focused on the United Kingdom (UK) noted that the published literature for emergency planning is dominated by event reports [11]. This can be problematic in that outcomes and conclusions may not be generalizable to other populations, settings, exposures or interventions [12-14]. Public health in general aims to be informed by the best available evidence and there is an appetite to have more rigorous forms of evidence to inform PHEP practice [12,16,17].

While the PHEP knowledge base is dominated by event reports and narrative reviews [11], PHEP research of a more analytical nature has been undertaken in some recent emergencies [10]. This review focuses on primary and analytical research in PHEP in order to better inform research priorities for the field. Our objectives in undertaking this scoping review are therefore twofold: first, to develop a thematic map of the PHEP evidence base, focused on primary and analytical research; and second, to use this map to inform and guide an understanding of knowledge gaps relevant to research and practice in PHEP.

## Methods

### Approach

We conducted a scoping review of published research literature in the field of public health emergency preparedness. The scoping review methodology is particularly relevant to fields which have an emerging and diverse knowledge base, which makes the method well-suited to the PHEP literature [18-20]. Scoping reviews may be undertaken for four reasons: to examine the extent, range and nature of research activity; to determine the value of undertaking a full systematic review; to summarize and disseminate research findings; and to identify research gaps in the existing literature [18]. This scoping review addresses three of the four reasons: to examine the extent, range and nature of research for PHEP; to summarize and disseminate research findings; and finally to identify existing gaps in the field that can be addressed through ongoing research. The six steps of the framework articulated by Arksey and O’Malley and further described by Levac et al. were applied in the review protocol [18-20]. As outlined by Levac et al., to maximize the rigor of the review, we implemented an iterative process throughout the six steps that incorporated knowledge from expert stakeholders.

### Step 1: Identifying the research purpose and scope of inquiry

The first purpose of the review is to create a map of the major themes in the PHEP evidence base to inform an understanding of the extent, range and nature of PHEP research. The second purpose is to identify potential knowledge gaps relevant to research and practice in PHEP by summarizing research findings and identifying existing gaps in the field. ‘Public health’ was defined for this review as the scope of public health activities relevant to Canadian public health agencies and departments; this scope includes activities such as surveillance, epidemiology, food and water safety, public information and communication, and laboratory services but notably excludes the provision of mass health care or hospital and institution-based emergency preparedness that may be included in PHEP in other countries [5]. Emergency preparedness research focused on all aspects of the emergency management cycle (prevention/mitigation, preparedness, response and recovery) was considered. An all-hazards approach was used including literature related to a range of hazards such as infectious disease, natural disasters, terrorism and technological events. The settings of complex humanitarian emergencies and conflict were out of scope. The focus was on peer-reviewed or grey literature and included studies of primary research, either quantitative or qualitative [21], and research examining standards or best practices (Table 1). Secondary research or knowledge syntheses such as literature reviews were excluded from the final group. Computer simulation studies were excluded for feasibility purposes and in order to focus on the scope of applied, real-world PHEP research.

**Table 1:** **Inclusion criteria for assessment of studies**

**Criterion 1**	Does the article specifically include the actions of Public Health (local, province/state or national level)?
**Criterion 2**	Does the article include public health actions in some aspect of emergency management (prevention/mitigation, preparedness, response, and/or recovery)?
**Criterion 3**	Does the article include an evaluation of public health actions during an emergency event (whether based on qualitative or quantitative data)
	OR does the article propose emergency management-related standards or best practices that have been derived from a process with clear methods?

### Step 2: Identifying relevant studies

A library specialist-assisted search was employed that addressed the concepts of public health, emergencies or disasters, emergency preparedness or emergency management and evidence or evaluation. The databases searched were Medline, Embase, BIOSIS, PsycInfo and EBSCO (CINAHL, Academic Search Premier, Health Business Elite, Environment Complete and SocINDEX) from 1998 to 2013 to include important events in the emergency management field such as SARS, 9/11 and Hurricane Katrina internationally and the 1998 ice storm in Canada (Additional file 1). In addition, reference lists of included articles were reviewed. Non-indexed sources of evidence were examined (grey literature) and a custom Google search was created to examine key government, agency and non-governmental organization websites for an international sample of organizations practicing in the PHEP field.

### Step 3: Study selection

Study selection was an iterative process that included screening first by title only, second by abstract, and finally, by full-text review. Initial search results were screened by title for their relevance to: (1) health, health consequences or health systems and (2) emergency preparedness or management actions. We conducted an initial pilot using 10% of studies reviewed by two reviewers to ensure consistency in applying the title screening criteria. After this pilot, the remaining initial search results were reviewed for relevance by title by one reviewer.

The second phase of study selection applied the inclusion criteria for relevance by abstract (Table 1). These criteria were developed in relation to the research purpose and refined based on consensus by members of the research team. The inclusion criteria were applied by two reviewers to the abstracts of title-screened search results and differences resolved by consensus.

Table 1 criteria were applied again to the screening of full text articles that were examined by two reviewers. A final group of selected articles was identified after full-text review; this group comprised the ‘primary research’ studies of interest. Themes identified from excluded simulation studies were cross-referenced with the themes from the final group of articles to ensure there were no notable elements or themes missed.

### Step 4: Charting the data

The descriptive-numerical summary approach described by Levac et al. was used for charting the data [19]. The data charting form was developed by consensus with the research team, piloted with two team members, and adapted iteratively in refining the final categories. The final table categories included were: research question/objective, category of study design (e.g., descriptive or analytical), specific study design, study population, intervention or exposure of interest, control group, study outcomes, study conclusions, hazard category, specific hazard, year of emergency event, scope of emergency event (e.g., local, provincial/state, federal or international support required), phase of emergency management cycle (e.g., prevention and/or mitigation, preparedness, response, recovery) [22], country of emergency event, country of study authors, and finally, additional notable elements or themes. If fields could not be completed or were not applicable for a given study, they were left blank. Assessment of quality of evidence beyond research design was out of scope for this review. The data charting form was completed by two reviewers and differences resolved by team consensus.

The category of study design allowed for the identification of a final group of articles that were primary research and evaluative or evidence-based in nature. This group was assessed using a thematic analysis approach which allowed for the capture of emergent themes in a more inductive method, rather than classifying the studies deductively based on previously described categories [19]. Thematic analysis was initiated by two reviewers and iteratively developed with the research team. Specifically, each reviewer examined papers by full-text and noted key themes for the study. Themes identified were discussed with the larger research team and noted in the final data charting form based on consensus.

### Steps 5 and 6: Collating, summarizing, reporting results and consultation

The consultation step is described by Levac et al. as essential and adding methodological rigor [19]. The objectives of the consultation phase were to share preliminary findings with stakeholders as knowledge translation, validate the evidence map of themes, and identify additional sources of information, meaning and applicability [18,19]. Collating and summarizing results was therefore done in phases related to these objectives. In the first phase of data collation and summary, numerical or quantitative in nature were summarized using Microsoft Excel™ 2010. The key themes from the final group of primary research studies were used to create groupings of similar studies by theme and summarized in table format. These initial results and themes were sent to stakeholders to elicit their feedback through a survey.

For the consultation step process, we used a sample of stakeholders invited to participate in a working group meeting funded by a Canadian Institutes of Health Research (CIHR) Planning Grant awarded to conduct a scoping review and engage stakeholders in setting research priorities for PHEP. The sample was identified in a purposive manner to be international and interdisciplinary in nature and represent a diversity of perspectives from within PHEP as well as closely related fields responsible for working with the public health system in emergencies. Participants represented the fields of public health, health care, emergency medical services, government, and were distributed across research, academic and policy environments. The number of participants was 26. Consultation was carried out via electronic survey administered using FluidSurveys™. Ethics approval was obtained by the Public Health Ontario Ethics Review Board. For each theme, the survey participants were asked whether additional evaluative literature exists, and if they were aware of any gaps relating to practice, based on their knowledge and expertise. In addition, participants were asked to note any additional themes or topics not captured in the review (Additional file 2).

The second phase of data collation and summary involved analysis of the results from the survey. Dichotomous (yes/no) responses were summarized using Microsoft Excel™ 2010. Qualitative data from open-ended questions were coded and summarized based on themes and relevant sub-themes. The results of the survey for suggested additional studies were assessed for inclusion using the same criteria from Table 1 to determine whether they met consistent criteria for inclusion in the review; while all additional suggested themes were included in the final list of themes.

The last aspect of consultation occurred during an in-person working group meeting with the stakeholders. The themes resulting from the review and survey were presented to participants who were given the opportunity to offer final comment and confirm the themes and sub-themes.

## Results

### Study selection and characteristics of the studies

**Figure 1:**
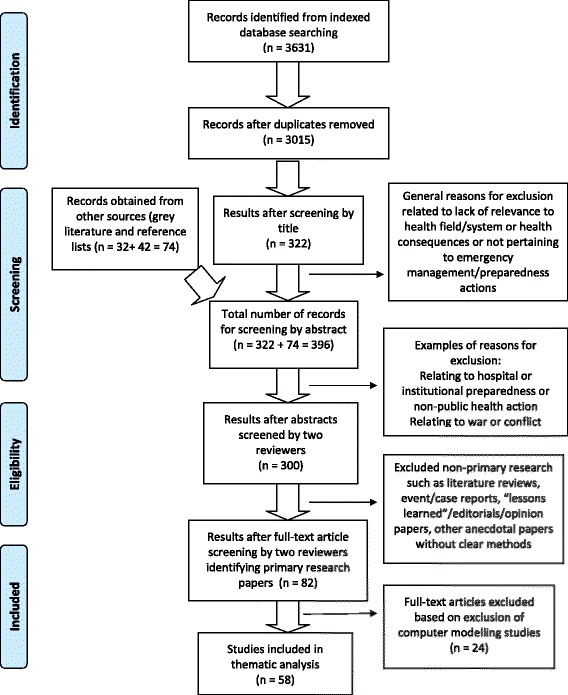
Flow diagram of pre-consultation article selection process.

The total number of citations retrieved through the search of indexed databases after duplicates removed was 3015. A flowchart of the article selection process is displayed in Figure 1. Three hundred articles met our criteria after title and abstract screening including additional sources from reference lists and grey literature. Reasons for exclusion of citations were predominantly related to emergency preparedness or management activities that did not meet the criteria of public health actions, based on the established definition. For example, hospital-based preparedness; mass casualty preparedness, triage or health care; and preparedness for institutions such as long-term care.

**Figure 2:**
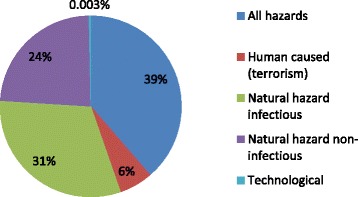
Types of hazards for studies identified in scoping review. 1. All hazards: dark blue, 2. Human-caused (terrorism): red, 3. Natural hazard infectious: green, 4. Natural hazard non-infectious: purple, 5. Technological: light blue.

Data charting by descriptive numerical summary was undertaken for the 300 articles that were selected after abstract screening. The types of disaster were: all hazards or disaster type unspecified (39%); infectious natural hazards (31%); and non-infectious natural hazards or natural disasters (24%) (Figure 2).

The largest proportion of articles related to the response phase of the emergency management cycle (42%) and the second largest to the preparedness phase (34%). The prevention/mitigation phase accounted for 21%, while recovery was represented by only 3% of articles. The majority of studies were published by United States (US)-based authors (197, 66.3%), with Canada ranking second (23, 7.7%). The largest number of emergency events described in the published literature was from the US (126, 42%), with the second largest from Canada (18, 6.0%).

**Figure 3:**
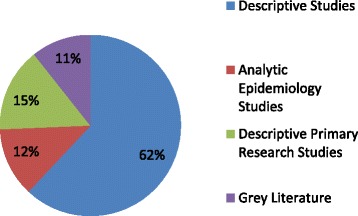
Types of study designs in scoping review. 1. Descriptive studies: dark blue, 2. Analytic epidemiology studies: red, 3. Descriptive primary research studies: green, 4. Grey literature: purple.

The majority of studies, 186 (62%), were non-primary research or descriptive articles. Thirty-seven (12%) were analytic epidemiology studies and 45 (15%) were descriptive primary research studies, such as surveys (Figure 3). Of the group of non-primary research or descriptive articles, 50% were emergency event-related descriptions such as “lessons learned”, event reports or case studies; 37% were literature reviews that did not employ systematic review methodology; 6% were commentaries; 3% were systematic reviews and another 3% were practice guidelines. A final 1% consisted of media or magazine reports that were captured in the search.

Eighty-two primary research and analytic epidemiology studies were selected for further review; these were reduced to 58 articles after exclusion of computer simulation studies. The final group of 58 articles related specifically to the research question. No grey literature met the criteria of being evidence-based or evaluative in nature. Within the final group, the most common study design was survey methodology (60%) including cross-sectional population surveys, surveys using qualitative methods such as key informant interviews, and surveys conducted after training exercises as an evaluation tool. The cohort studies were largely retrospective administrative database analyses. Studies with an evaluation purpose were largely evaluations of a process change or of training.

### Identification of themes

#### Phase 1

The first iteration of thematic analysis resulted in the identification of ten major themes (Table 2). The key findings for the initial themes are described below, with additional detail provided in Additional file 3.

**Table 2:** **Initial themes emerging from scoping review on evidence-based knowledge for PHEP**

	**Emerging theme from the evidence-based literature for PHEP**	**Subthemes, if applicable**	**No. of studies (total)**
**1**	Attitudes and beliefs	Willingness to respond	3
**2**	Capacity assessment and capacity-building	-	7
**3**	Collaboration and system integration	-	4
**4**	Communicable disease control		2
**5**	Communication	Externally-facing (public)	4
		Internally-facing (system)	3
		Vulnerable or high-risk populations	1 (8)
**6**	Education, training and exercises	Public health practitioner	7
		Clinician	1
		Leadership	1 (9)
**7**	Public health considerations for sheltering and evacuation	-	2
**8**	Quality improvement and performance standards	-	5
**9**	Surveillance, epidemiology and public health information	Unspecified	8
		Rapid needs assessments	4
		Risk assessment	1 (13)
**10**	Vulnerable populations	Vulnerability assessment	2
		High-risk populations	3 (5)
**TOTAL**			58

*Attitudes and beliefs* [23-25]: A key sub-theme in the literature relating to attitudes and beliefs is the willingness of workers to respond to a public health emergency. In identified studies, willingness to respond to particular emergencies varies by type of disaster and practice setting [23,25]. In addition, studies concluded that willingness as an attitude is important to consider in emergency response training [23,24].

*Capacity assessment and capacity-building* [26-31]: A study in this theme reports that surveys at the local health department level can provide information to guide development of capacity [31]. Evaluative information that relates to emergency preparedness and response can inform a deeper understanding of the capabilities important to preparedness [27]. Another study notes the importance of funding on the preparedness capacity of local public health [29].

*Collaboration and system integration* [32-35]: Several studies highlight the importance of collaboration and integration in relation to public health emergencies. Collaborating on exchanging plans and protocols was identified as an important and feasible goal for agencies [34]. Roles exist in the health system that can facilitate linkages between health care and public health settings; for example, epidemiologists that link with hospitals [35]. Newer methodologies, like network analysis, present opportunities for examining the role of inter-agency networks in promoting prepared public health systems [33].

*Communicable disease control* [36,37]: This theme is a well-understood core function of public health. Studies on this theme found relating to PHEP research indicate the utility of specific emergency plans to inform public health strategies such as vaccination and examining the role of interventions such as school closure as mitigating measures in responding to a public health incident [36,37].

*Communication* [38-45]: The third highest number of studies was identified in this theme. Sub-themes noted for this theme relate to the focus of communication as externally-facing (to the public), internally-facing (within the system), and high-risk populations. Externally-facing communication examined aspects of the adoption of public health guidance by the public. For example, concern or risk perception may not translate automatically to higher uptake of guidance and skepticism regarding institutions may influence public uptake of recommended actions [39,45]. Internally-facing communication that occurs within the system included findings that stakeholders have preferences on how information is received and note the importance of trust in the agency delivering the message [40,43]. Principles of trust are also important when communicating with high-risk populations [46].

*Education, training and exercises* [47-55]: This theme contained the second highest number of studies. Sub-themes were developed that related to the audience of the education or training intervention such as public health practitioner, clinician and leader. Public health practitioner studies found that blended learning approaches had good outcomes for both satisfaction and knowledge [52]. Studies also found that criteria or tools can be used to facilitate evaluation of training and exercises [51,53]. Leadership-specific training was reported to result in increased overall knowledge and positive change [55].

*Public health considerations for sheltering and evacuation* [56,57]: Studies identified in this theme describe the importance of the planning process to address the health needs of sheltered populations [57]. In addition, public health training in shelter operations was noted as a gap [56].

*Quality improvement and performance standards* [58-62]: This theme included studies that explore models for improving public health preparedness as well as frameworks and tools that can be used to measure preparedness and promote quality improvement [61,62]. Processes for team debriefing were also found to be helpful to evaluate performance [58].

*Surveillance, epidemiology and public health information* [63-75]: This theme was associated with the largest proportion of studies (13/58, 22%). Within this theme, methodologies applied in epidemiological data collection and analysis were identified. Specific sub-themes included studies on rapid needs assessments, which is a method that can provide valuable information to authorities on the needs and health status of affected communities [65,70,75]. Risk assessments using surveys can provide information about exposure risk to populations [69]. In addition, other studies described the use of a diversity and adaptability of data sources that can be applied to public health surveillance; for example, medical dispatch data and mobile phone data [63,73].

*Vulnerable populations* [76-80]: Sub-themes identified for this theme included methods for vulnerability assessment, such as heat vulnerability index, and high-risk populations in general. For high-risk populations, the challenges of identifying and prioritizing vulnerable populations were noted and strategies for integrating diverse communities into emergency planning and response were explored [76,77].

#### Phase 2

A list of the initial themes along with summarized key findings from the relevant articles was disseminated to stakeholders. Of 26 participants invited to participate in the survey, the response rate was 62%.

Four additional themes were noted by stakeholders: ethics, gender considerations, psychosocial considerations, resilience and planning. Ethics, gender, resilience and psychosocial considerations had been noted in the evidence reviewed but had not been captured as themes. Planning was a theme that was identified as important based on experience, rather than literature. In addition, wording refinements for themes were suggested based on participants’ expertise. For example, “high risk” or “at-risk” populations was suggested instead of “vulnerable populations”. Although the majority of the studies were conducted in the US, 88% of survey respondents felt the themes were applicable to PHEP in Canada.

There were 29 total additional citations that were suggested by stakeholders as potential evidence for inclusion in the review. It was determined by the research team that 2 additional articles would have met the inclusion criteria applied in the review [81,82]. Reasons for exclusion included: articles that were not related to public health actions (E.g. hospital or emergency responder actions), not representing primary research, or outside the date criteria. The additional articles and survey results were used to revise the original PHEP themes. A number of themes were cross-cutting in nature, while others stood on their own. The synthesized list of themes included 4 cross-cutting themes and 12 stand-alone themes (Table 3).

**Table 3:** **Final group of stand-alone themes emerging for the PHEP evidence base**

	**Theme**	**Sub-theme, if applicable**	**Cross-cutting themes**
**1**	Attitudes and beliefs	Willingness to respond	EQUITY
**2**	Capacity assessment and capacity-building	Community capacity	GENDER CONSIDERATIONS
		Multi-sectoral capacity	HIGH RISK OR AT-RISK POPULATIONS
		Organizational capacity	RESILIENCE
**3**	Collaboration and system integration	Including:	
		○ Acute care	
		○ Long-term care	
		○ Community care	
		○ Primary care	
		○ Emergency medical services	
**4**	Communicable disease control		
**5**	Communication	Externally-facing (public)	
		Internally-facing (health or government system)	
		Social media (cuts across subthemes)	
**6**	Education, training and exercises	Clinician	
		Leadership	
		Public health practitioner	
**7**	Ethical considerations		
**8**	Planning		
**9**	Psychosocial impacts of emergencies	Community	
		Health workers	
**10**	Public health considerations for sheltering and evacuation	Functional needs assessments	
**11**	Quality improvement and performance standards	Measurement and metrics	
**12**	Surveillance, epidemiology and public health information	Rapid needs assessments	
		Risk assessment	

In the final stage of stakeholder consultation, the synthesized and revised results consisting of cross-cutting and stand-alone themes were presented to the survey participants in a face-to-face format. This step resulted in the confirmation of the themes as presented in Table 3 without further suggested revisions.

### Knowledge gaps

This review aimed to identify knowledge gaps for PHEP relevant to research and practice in the field. Primary research from non-US settings was identified as a general gap, consistent with previous reviews [11]. The results of the survey questions pertaining to participants’ sense of knowledge gaps, for the initial ten themes, is described below with additional detail provided in Additional file 4.

*Attitudes and beliefs:* Gaps pertained to the ethical values involved in assessing willingness to respond; the importance of psychosocial supports to foster willingness to work; and volunteer characteristics.

*Capacity assessment and capacity-building*: Gaps for this initial theme related to building capacity to address a need for psychosocial supports for workers and building capacity for hospital surge. General capacity-building also noted the importance of poverty reduction in mitigating the impact of emergencies on populations.

*Collaboration and system integration*: Gaps pertaining to this theme related to cross-jurisdictional collaboration as well as across sectors of the health system.

*Communicable disease control*: A gap that was noted relating to SARS was the labour issues involved for front line health care workers in the setting of communicable disease outbreaks.

*Communication*: Communication as a strategy to promote integration with other sectors such as primary care was noted as a gap. In addition, emerging information technologies such as social media have had limited exploration to date.

*Education, training and exercises*: A number of gaps were identified in the survey pertaining to this initial theme. These ranged from specific capabilities such as Incident Management System implementation to more complex concepts such as flexibility and emergency decision-making for public health practitioners.

*Public health considerations for sheltering and evacuation*: Gaps in this initial theme related to understanding the logistics and implementation challenges for public health measures such as evacuation. In addition, the potential for public health to benefit from skillsets found in other disciplines, which could promote improved public preparedness levels.

*Quality improvement and performance standards*: Within this theme, the challenge of understanding the components of preparedness was noted. Psychosocial supports was again noted as a gap in this theme.

*Surveillance, epidemiology and public health information*: Elements of integration to promote public health information were noted, such as the interfaces of public health with primary and emergency care. The value of technologies such as electronic health records was described as a gap in relation to this theme. The concept of resilience was also raised as a gap.

*Vulnerable populations*: A number of gaps pertaining to vulnerable populations were noted. The ethical values and considerations inherent in exploring issues of “vulnerable” populations were described. In addition, methods for promoting community engagement for public health preparedness were noted and for addressing the needs of specific populations and potential vulnerabilities.

The following specific quotes relate to qualitative data collected on knowledge gaps, with relevant associated theme:

*“What are the main methods and coping strategies used by individuals, stakeholders and their organizations to get back to normal life?”* (**resilience**);

*“How do we answer the frequent question: “Are we prepared?”* (**quality improvement and performance standards**);

*“There is more focus on recovery needed in the literature, whether physical or psychosocial impacts”* (**resilience, psychosocial impacts, capacity assessment and capacity building**);

*“There is a need for collaboration and networking across disciplines to scope beyond PHEP/public health”* (**collaboration and system integration**).

Based on thematic analysis and synthesis of the initial themes with survey results, we identified five key knowledge gaps that were important areas of interest to stakeholders but where evidence is sparse or non-existing. These are depicted along with the cross-cutting themes identified from the consultation (Table 4).

**Table 4:** **Knowledge gaps identified for PHEP**

	**Theme**	**Description of relevant knowledge gap**	**Cross-cutting themes**
**1**	Attitudes and beliefs	Behavioural aspects relevant to PHEP E.g. Relating to emergency decision-making and communication strategies	EQUITY
**2**	Collaboration and system integration	Integration of public health/PHEP with other sectors and components of the health system	GENDER CONSIDERATIONS
**3**	Communication	Use and application of emerging technologies such as social media and electronic health records	HIGH RISK OR AT-RISK POPULATIONS
**4**	Quality improvement and performance standards	Measurement of performance or capacity	RESILIENCE
**5**	Resilience	Public health roles in recovery and in returning individuals and systems to normal life after an emergency	

## Discussion

Previous reviews of PHEP evidence base documented the largely descriptive and anecdotal nature of the literature and found it challenging to draw conclusions as a result [7,11]. Rigorous and analytical research needs to be conducted to strengthen the PHEP evidence base to respond to practice and policy-related questions for the field [9,10]. This review focused on the body of primary research that has been conducted in the PHEP field and aimed to map important themes, for the purpose of furthering understandings of existing PHEP science and revealing potential knowledge gaps for future research. This scoping review is novel for the PHEP field in that it utilized published scoping review methodology with the identification of themes as they emerged, without applying a pre-existing framework [19]. In addition, the study included expert stakeholder consultation to validate and confirm the themes identified by the review, a knowledge translation step that is sometimes omitted in scoping reviews but considered essential [19].

The iterative identification of themes using the literature and survey methods resulted in the recognition of important cross-cutting themes for PHEP. To our knowledge, this is the first time that the cross-cutting nature of some PHEP themes has been documented. Based on these results, consideration of the themes of at-risk populations, gender and equity should be recognized as integral to planning and implementing PHEP research [83]. Although these themes may be viewed as similar they each have unique characteristics: at-risk populations vary depending on the hazard and risk, gender differences cut across virtually all population health issues, and equity speaks more broadly about disparities that may pre-exist within a given community. Resilience was the fourth cross-cutting theme [84]. Resilience is described as the intrinsic capacity of individuals, populations and infrastructure to resist and rebound from shocks and to then rebuild to a stronger state [84]. In national-level emergency management policy, resilience is established as the overall objective of all-hazards preparedness and capacity-building activities in order to reduce the impact of emergencies on a given population [22,85]; hence its inclusion in this category.

In addition to cross-cutting themes, the stand-alone themes with the largest number of papers included in the review were Surveillance, Epidemiology and Public Health Information, Communication and Education, Training and Exercises. Surveillance, Epidemiology and Public Health Information was the theme with the largest number of studies. This is not surprising given the integral nature of surveillance and epidemiology to public health, as well as the manner in which quantitative data are used for analytic processes to assess risk or measure outcomes of public health emergency events. Efforts in the US to identify key domains for PHEP are consistent and have similarly identified ‘health surveillance’ [86]. Communication is an area that has been noted to be problematic in post-emergency event assessments; thus, it is positive that research is emerging under this theme [6,87]. The social behavioural facets of communication relating to PHEP have recently been explored in the literature. For example, concepts of trust and preferences for certain characteristics of messaging highlight the contextual aspects that are emerging from PHEP communication research [40,43,45]. Important knowledge gaps were identified for communication in PHEP relevant to the use of emerging technologies. Education, Training and Exercises was another theme in which preliminary study on approaches to training for ‘hard skills’ such as technical skills and ‘soft skills’ such as leadership has been undertaken [53,54]. Nonetheless, attention to training as it relates to emergency decision-making in public health was highlighted as a gap in this review.

The complexity of PHEP is reflected in the knowledge gaps that emerged. For example, Collaboration and System Integration sub-themes pertain to multiple health-care stakeholders. Multi-sectoral partnerships and engagement has also been noted as important domains for PHEP operations in the US [86]. Opportunities for generating knowledge to examine complexities such as system integration could address knowledge gaps and inform operational activities and policy. Another notable knowledge gap relates to the theme of Quality Improvement and Performance Standards. Multiple publications have noted the importance of quality improvement and development of standards or metrics for PHEP [5,9,88]. Despite this, little research was identified that has empirically examined the development of standards and metrics. Research in PHEP that advances empirical knowledge in this area would be valuable.

This review has several limitations. First, this study specifically focused on research that was relevant to the actions and activities of public health as defined for the Canadian setting. The findings therefore may not translate to other countries that have differing scope of public health responsibilities. As was noted in the emergent themes, the importance of integration and collaboration within the health system is key for PHEP, and it is possible that this review did not capture the extent of public health connectedness to the rest of the health system, as found in the literature. This focus on public health was carefully defined, however, with the purpose of identifying themes and relevant knowledge gaps that specifically pertain to public health agencies and departments. Second, as with all systematic reviews, there is a possibility that literature was missed, which may have resulted in a lack of recognition of key themes or gaps. We used a rigorous search strategy for the indexed and grey literature to minimize this. In addition, we conducted stakeholder consultation in order that experts in the field would provide information and contribute knowledge that was missing. As a result of this iterative approach, additional articles were included in the final thematic analysis. Third, computer simulation studies were excluded due to scope of the current study, with a focus on the field-derived primary research studies. An attempt to account for themes that resulted from data-charting of this group was done and compared with the final emergent themes. In general, these studies pertained to vaccination and outbreak management, which were captured under themes of Communicable Disease Control or Surveillance, Epidemiology and Public Health Information. Finally, quality of research studies was not assessed beyond research design in this review and for this reason, the themes identified in the literature and the knowledge gaps do not take into account specific methodological strengths and weaknesses of the included studies. While quality assessment is ideal to include, we addressed the varying quality in the PHEP literature by focusing on identifying a group of articles that did not include event reports or ‘lessons learned’ commentaries, and focused on primary research studies.

A question that has been posed in a review of the emergency planning literature focused on the UK is whether “the traditional hierarchy of evidence applied to clinical research is appropriate in this field” [11]. The challenge of using the traditional evidence hierarchy to inform evidence-based decision-making in health has been raised outside the PHEP field [21]. The hierarchical approach to evidence is quantitatively oriented and does not elaborate on how context may modify the relevance of evidence [21]. Upshur et al. suggest that an inclusive definition of evidence includes both quantitative research that involves measurement as well as qualitative research that examines context and meaning [21]. This review included the breadth of evidence described in this taxonomy, and identified qualitative research in PHEP that attempts to capture meaning. The themes identified in this review for PHEP would support that both quantitative and qualitative methodological approaches will be valuable in generating knowledge to understand the complexities of gaps such as communication, collaboration and integration, and attitudes and beliefs. However, as noted by Upshur et al., such research will benefit from having a sufficient theoretical base that will allow it to have solid ‘scientific’ standing in the eyes of proponents of evidence-based practice. This is important given the high proportion of anecdotal event reports which share experiential and contextual knowledge but generally lack a sufficient theoretical base. In addition, there are recent studies highlighting that participatory and community-based approaches to research may have an important role in addressing some of the cross-cutting and stand-alone themes noted in this review [89,90]. Participatory methods hold promise for promoting inclusiveness across a community and examining the meaning that may underlie the complexities of the emergency [89]. These methods also model a collaborative approach that may promote resilience, enabling both research and community-building, thereby fostering the translation of knowledge to action [89,91].

## Conclusions

Global health threats, which include emerging infectious diseases, technological events, and natural disasters, appear to be increasing in frequency and impact. This scoping review identifies the key cross-cutting and stand-alone themes that characterize the current state of the evidence base of primary research in PHEP. The identification of themes and related knowledge gaps represents a leaping-off point from which to identify research priorities and inform the further generation of practice-oriented research to advance PHEP. Based on the diversity of themes and knowledge gaps identified, collaboration in inter-disciplinary teams such as was utilized in the consultation step of this review and an inclusive definition of evidence, will be valuable in developing research that is both rigorous and relevant to the field.

## Additional files

Additional file 1:
**Example of Search Strategy as Applied in Medline.**
Additional file 2:
**Example of Survey Questions.**
Additional file 3:
**Summary table of key findings from studies selected by initial theme.**
Additional file 4:
**Summarized survey results for applied knowledge gaps by initial theme.**

## Abbreviations

CIHR: Canadian Institutes of Health Research
PHEP: Public health emergency preparedness
SARS: Severe acute respiratory syndrome
UK: United Kingdom
US: United States
