# Supplementary material for: The evidence base of primary research in public health emergency preparedness: a scoping review and stakeholder consultation
Source: BMC Public Health. 2015 Apr 28;15:432. doi: 10.1186/s12889-015-1750-1 (PMC4415223; doi:10.1186/s12889-015-1750-1)
Supplement: Additional file 1: — Example of Search Strategy as Applied in Medline. [file 12889_2015_1750_MOESM1_ESM.docx]

**Additional file 1: Example of Search Strategy as Applied in Medline**

| # | Searches |
| --- | --- |
| 1 | (("public health" adj1 (practice or administration)) or "preventive medicine").mp. |
| 2 | ((natural adj1 (disaster? or hazard?)) or (hurricane? or flood$ or typhoon? or earthquake$ or fire? or cyclon$ or heatwave? or freezing or ((ice or snow or lightning) adj1 storm?) or blizzard? or "heat wave" or (extreme adj1 (temperature? or heat or cold)) or tsunami? or "tidal wave")).mp. |
| 3 | (firesetting or arson or explosion? or bomb$ or (explo$ adj1 device?) or blackout? or brownout? or ((power or equipment) adj1 (loss or failure)) or radioactive or radiation or (nuclear adj1 (disaster or meltdown or catastrophe or fail$))).mp. |
| 4 | (epidemic? or pandemic? or outbreak? or influenza).mp. |
| 5 | (terroris$ or bioterroris$ or ((chemical or biological) adj1 warfare)).mp. |
| 6 | (riot$ or (civil adj1 (disorder? or defense or unrest))).mp. |
| 7 | ((emergency adj1 (preparedness or response or management)) or (disaster adj1 (plan$ or preparedness or mitigation or recovery or cycle or medicine or resilience)) or (risk adj1 (assessment or communication or management)) or "organizational structures" or (incident adj1 (command or management)) or "critical decision making" or interoperability).mp. |
| 8 | or/2-7 |
| 9 | public health/ or preventive medicine/ or public health practice/ or public health administration/ |
| 10 | disasters/ or emergencies/ or mass casualty incidents/ |
| 11 | cyclonic storms/ or droughts/ or floods/ or tidal waves/ or tsunami/ or snow/ or rain/ or avalanches/ or volcanic eruptions/ or earthquakes/ or landslides/ or fires/ or ice/ or tornadoes/ or extreme cold/ or extreme heat/ or lightning/ or cold temperature/ or hot temperature/ or wind/ |
| 12 | firesetting behavior/ or explosions/ or blackout/ or equipment failure/ or radioactive hazard release/ |
| 13 | epidemics/ or pandemics/ or disease outbreaks/ or influenza/ |
| 14 | terrorism/ or bioterrorism/ or chemical terrorism/ or "september 11 terrorist attacks"/ |
| 15 | riots/ or civil disorders/ or civil defense/ |
| 16 | emergency preparedness/ or emergency response/ or emergency management/ or disaster planning/ or disaster medicine/ or disaster resilience/ or risk communication/ or organization structures/ or incident command/ or incident management/ or critical decision making/ or interoperability/ |
| 17 | (evidence or evaluat$ or myth$ or "lessons learned" or "lessons learnt" or AAR or "after action review" or "after action reviews").mp. |
| 18 | Evidence-Based Medicine/ |
| 19 | Evidence-Based Practice/ |
| 20 | Evaluation Studies as Topic/ |
| 21 | ((disaster adj1 (vulnerability or "risk reduction")) or (social adj1 (vulnerability or resilience))).mp. |
| 22 | or/17-20 |
| 23 | 8 or 21 |
| 24 | or/10-16 |
| 25 | 23 or 24 |
| 26 | 1 or 9 |
| 27 | 25 and 26 and 22 |
| 28 | limit 27 to ((english or french) and last 15 years) |
