# Supplementary material for: The evidence base of primary research in public health emergency preparedness: a scoping review and stakeholder consultation
Source: BMC Public Health. 2015 Apr 28;15:432. doi: 10.1186/s12889-015-1750-1 (PMC4415223; doi:10.1186/s12889-015-1750-1)
Supplement: Additional file 2: — Example of Survey Questions. [file 12889_2015_1750_MOESM2_ESM.docx]

**Additional file 2: Example of Survey Questions**

1. We are interested in documenting the regions, jurisdictions and roles of meeting participants. This data will not be presented or used in any identifying way, nor linked to survey responses. Please identify your region and setting below:
2. Region: [Canada, USA, Europe, other]
3. Employment jurisdiction: [national, provincial/state, municipal, university, private sector, other]
4. Role: [researcher, knowledge user/practitioner, both researcher and knowledge user, other]

The next ten questions pertain to each theme identified in the current scoping review. The questions ask, by theme, in part (a), whether you identify and can share any additional evidence-based or evaluative literature; in part (b), whether you identify any additional knowledge areas or gaps in current practice that have not been captured in the literature.

1. Based on your knowledge and expertise:
   1. Is there any **evidence-based or evaluative** literature that has **NOT** been included in this review for the theme of **surveillance, epidemiology and public health information**?
      - - Y/N
        - If yes, please provide us with the source and title of that information below
   2. What additional themes, topics or notable gaps related to public health emergency preparedness **practice** for the theme of **surveillance, epidemiology and public health information** are not captured in this review?
2. Based on your knowledge and expertise:
   1. Is there any **evidence-based or evaluative** literature that has **NOT** been included in this review for the theme of **vulnerable populations, whether pertaining to vulnerability assessment or high-risk populations**?
      - - Y/N
        - If yes, please provide us with the source and title of that information below
   2. What additional themes, topics or notable gaps related to public health emergency preparedness **practice** for the theme of **vulnerable populations, whether pertaining to vulnerability assessment or high-risk populations** are not captured in this review?
3. Based on your knowledge and expertise:
   1. Is there any **evidence-based or evaluative** literature that has **NOT** been included in this review for the theme of **education, training and exercises, whether pertaining to public health practitioner, clinician or leadership training**?
      - - Y/N
        - If yes, please provide us with the source and title of that information below
   2. What additional themes, topics or notable gaps related to public health emergency preparedness **practice** for the theme of **education, training and exercises, whether pertaining to public health practitioner, clinician or leadership training** are not captured in this review?
4. Based on your knowledge and expertise:
   1. Is there any **evidence-based or evaluative** literature that has **NOT** been included in this review for the theme of **communications, whether pertaining to externally-facing (public), internally-facing (system) or vulnerable population considerations in communications**?
      - - Y/N
        - If yes, please provide us with the source and title of that information below
   2. What additional themes, topics or notable gaps related to public health emergency preparedness **practice** for the theme of **communications, whether pertaining to externally-facing (public), internally-facing (system) or vulnerable population considerations in communications** are not captured in this review?
5. Based on your knowledge and expertise:
   1. Is there any **evidence-based or evaluative** literature that has **NOT** been included in this review for the theme of **collaboration and system integration**?
      - - Y/N
        - If yes, please provide us with the source and title of that information below
   2. What additional themes, topics or notable gaps related to public health emergency preparedness **practice** for the theme of **collaboration and system integration** are not captured in this review?
6. Based on your knowledge and expertise:
   1. Is there any **evidence-based or evaluative** literature that has **NOT** been included in this review for the theme of **quality improvement and performance standards**?
      - - Y/N
        - If yes, please provide us with the source and title of that information below
   2. What additional themes, topics or notable gaps related to public health emergency preparedness **practice** for the theme of **quality improvement and performance standards** are not captured in this review?
7. Based on your knowledge and expertise:
   1. Is there any **evidence-based or evaluative** literature that has **NOT** been included in this review for the theme of **capacity assessment and capacity-building**?
      - - Y/N
        - If yes, please provide us with the source and title of that information below
   2. What additional themes, topics or notable gaps related to public health emergency preparedness **practice** for the theme of **capacity assessment and capacity-building** are not captured in this review?
8. Based on your knowledge and expertise:
   1. Is there any **evidence-based or evaluative** literature that has **NOT** been included in this review for the theme of **attitudes and beliefs, including willingness to respond**?
      - - Y/N
        - If yes, please provide us with the source and title of that information below
   2. What additional themes, topics or notable gaps related to public health emergency preparedness **practice** for the theme of **attitudes and beliefs, including willingness to respond** are not captured in this review?
9. Based on your knowledge and expertise:
   1. Is there any **evidence-based or evaluative** literature that has **NOT** been included in this review for the theme of **public health considerations for sheltering and evacuation**?
      - - Y/N
        - If yes, please provide us with the source and title of that information below
   2. What additional themes, topics or notable gaps related to public health emergency preparedness **practice** for the theme of **public health considerations for sheltering and evacuation** are not captured in this review?
10. Based on your knowledge and expertise:
    1. Is there any **evidence-based or evaluative** literature that has **NOT** been included in this review for the theme of **communicable disease control**?
       - - Y/N
         - If yes, please provide us with the source and title of that information below
    2. What additional themes, topics or notable gaps related to public health emergency preparedness **practice** for the theme of **communicable disease control** are not captured in this review?
11. Based on your knowledge and expertise, are there any additional **themes or topics** that have not been captured in the current scoping review on evidence-based knowledge for public health emergency preparedness?
    1. Y/N
    2. If yes, please provide us with the source and title of that information below.
12. Which of the identified themes or topics presented in this scoping review are **applicable** to the **Canadian** context?
    1. All
    2. None
    3. Some – Please specify:
    4. Don’t know
13. Do you have any further comments or recommendations that can be used to guide our meeting on April 8^th^ and 9^th^ on setting research priorities for public health emergency preparedness in Canada?
