# Supplementary material for: The evidence base of primary research in public health emergency preparedness: a scoping review and stakeholder consultation
Source: BMC Public Health. 2015 Apr 28;15:432. doi: 10.1186/s12889-015-1750-1 (PMC4415223; doi:10.1186/s12889-015-1750-1)
Supplement: Additional file 3: — Summary table of key findings from studies selected by initial theme. [file 12889_2015_1750_MOESM3_ESM.docx]

**Additional file 3: Summary table of key findings from studies selected by initial theme**

| Theme | Subthemes, if applicable | Key findings |
| --- | --- | --- |
| Attitudes and beliefs | Willingness to respond | - Barriers to public health system functioning during emergencies include attitudes such as willingness, which can vary by type of disaster and practice location, whether urban or rural (DeSimone, 2009; Barnett, 2012) - Attitudes such as willingness are important to consider in training in emergency response (Barnett, 2009 and 2012) |
| Capacity assessment and capacity-building |  | - Evaluations of planning, preparing, and responding enhance our understanding of preparedness and capabilities (Davis, 2007) - Surveys of health departments can capture data on resources and capacity required to manage outbreaks (Porten, 2006) - Funding should be provided to local health departments in order for personnel to achieve expected outcomes, increase training and professional development, increase communication and collaborations including public health and in emergency preparedness (Hyde, 2006) |
| Collaboration and system integration |  | - Public health epidemiologists effectively link public health agencies and hospitals to enhance syndromic surveillance, communicable disease management, and public health emergency preparedness and response (Markiewicz, 2012) - The organizational dimensions of public health preparedness depend on a complex mix of individual organizational characteristics, inter-agency relationships, and institutional environmental factors (Moore, 2006) - The need to collaborate on and exchange plans and protocols among agencies was identified as the most important and most feasible across all groups (Olson, 2005) - Methods such as network analysis present options to study the role of inter-agency networks in the development of prepared public health systems (Moore, 2006) |
| Communicable disease control |  | - Emergency plans such as continuity of operations and pandemic influenza plans will allow the implementation of effective vaccination strategies in the pandemic setting (DiBiase, 2011) - School cycles indicate that school closure and other mitigation measures could be useful to mitigate future influenza pandemics (Chowell, 2011) |
| Communications | Externally-facing (public) | Adoption of public health advice may be related to considerations for communication and audience:   - High concern did not translate to higher compliance with recommendations possibly due to the low level of knowledge or economic barriers (Balkhy, 2010) - People actively engage in skeptical evaluation of government advice, particularly in terms of feasibility, credibility, and costs, which influence whether they adopt recommended actions (Teasdale, 2011) - Information alerts to the public may offer potential for early diagnosis of cases in an outbreak setting (Hiam, 2005) |
|  | Internally-facing (system) | - Potential gaps in communication include (Harris, 2007):   - The level of regular communication across local planners in different regions was low   - There was a shortage of connections to private-sector individuals - In communicating to frontlines clinicians during a public health emergency, clinicians would prefer a single source of authoritative information and ability to easily recognize new information specific to their needs (Staes, 2011) - Effective communication during a public health emergency depends on the provision of clear messages and close involvement of the affected community to ensure trust in agency delivering message (Blanchard, 2005) |
|  | Vulnerable or high-risk populations | - Social determinants are important and influence reception to disaster communications; trust in the source of information is also a key consideration for risk messaging (Taylor-Clark, 2010) |
| Education, training and exercises | Public health practitioner | - Blended learning that includes traditional face-to-face learning coupled with online learning had good outcomes for user satisfaction and knowledge (Chandler, 2008) - Evaluation of training and exercises could include considerations such as:   - A set of criteria covering command and control, communication, surveillance, sample testing (Gebbie, 2006)   - A tool implemented during tabletop exercises to identify public health capabilities in need of further improvement (Savoia, 2009)   - Self-assessment should be paired with objective measures, such as drills, exercises, and tests of knowledge (Kerby, 2005) - Audiences for training can include general public health staff, public health nurses or address particular topics such as legal preparedness for infectious disease emergencies (Quereshi, 2004; Savoia, 2009; Wang, 2008) |
|  | Clinician | - Public health can benefit from partnering with clinicians for training in areas such as proper selection and use of respirators for infection control (Alfano-Sobsey, 2006) |
|  | Leadership | - A focus on leadership training can result in increased overall knowledge score and positive change in skills (Wang, 2008) |
| Public health considerations for sheltering and evacuation |  | - Better public health training is recommended for sheltering operations (Brahmbhatt, 2009) - Planning is important to address health and health care needs of sheltered populations (Broz, 2009) |
| Quality improvement and performance standards |  | - Useful process evaluations may include team debriefing (Flabouris, 2004) - Frameworks and measurement tools may be useful to systematically evaluate performance for quality improvement goals (Sarpy, 2003; Parker, 2005) - A model for improving public health preparedness may be promising for improving public health performance which includes: aims and goals of public health departments; performance measures; strategies and ideas for changes; and the use of plan-do-study-act (PDSA) cycles; however, further efforts are needed (Lostein, 2008) |
| Surveillance, epidemiology and public health information | Unspecified | - Sources of data that are useful for public health surveillance in public health emergencies may include diverse sources such as:   - Medical dispatch data (Bassil, 2008)   - Syndromic surveillance data (Betancourt, 2007)   - Municipal data for mortality (Fan, 2010)   - Active lab-based surveillance system (Ghosh, 2008)   - Mobile phones for infectious disease surveillance info in a post-disaster setting (Yang, 2009) - Immediate health needs and longer-term health outcomes can be captured by cross-sectional and longitudinal surveys (Yzermans, 2005; Schnitzler, 2007) |
|  | Rapid needs assessments (RNAs) | - RNAs provided information to local and state authorities about types and magnitude of needs and health status of the affected communities (Chen, 2003; McNeil, 2006; Zane, 2010) - Emergency preparedness must be based on carefully conceived priorities, information, and communications, and improved capabilities must be developed to rapidly implement an emergency public health network (Chen, 2003) - A locally designed and deployed tool may provide population-based estimates of community needs or extent of event-related consequences (Groenewald, 2006) |
|  | Risk assessment | - Public health surveys may be useful to provide information on risk of exposures, such as nuclear (Maguire, 2010) |

| Vulnerable populations | Vulnerability assessment | - A developed heat vulnerability index may be a marker of *health* vulnerability in general (Reid, 2009 and 2012) |
| --- | --- | --- |
|  | High-risk populations | - A model for assessing capacity to integrate diverse communities into preparedness planning and response should include (Andrulis, 2011):  1. Engaging diverse communities in all aspects of emergency planning, implementation, and evaluation 2. Mitigating fear and stigma 3. Building organizational cultural competence 4. Enhancing coordination of information and resources  - It can be challenging to identify and prioritize vulnerable individuals (Abrahamson, 2009; Andrulis, 2011) - Vulnerable populations such as children may have greater emotional and behavioural consequences from emergencies such as terrorism (Stein, 2004) |
