# Supplementary material for: The evidence base of primary research in public health emergency preparedness: a scoping review and stakeholder consultation
Source: BMC Public Health. 2015 Apr 28;15:432. doi: 10.1186/s12889-015-1750-1 (PMC4415223; doi:10.1186/s12889-015-1750-1)
Supplement: Additional file 4: — Summarized survey results for applied knowledge gaps by initial theme. [file 12889_2015_1750_MOESM4_ESM.docx]

**Additional file 4: Summarized survey results for applied knowledge gaps by initial theme**

|  | **Initial themes** | **Knowledge gaps** |
| --- | --- | --- |
| **1** | Attitudes and beliefs | Convergent volunteers  Psychosocial supports  Ethics of willingness to respond |
| **2** | Capacity assessment and capacity-building | Reduction of poverty and health risks  Hospital surge capacity  Psychosocial supports for first responders and receivers  Multi-sectoral capacity essential to public health functions in a disaster  Resilience |
| **3** | Collaboration and system integration | Public health and community EOCs  Hospital surge capacity & public health management of mass casualty event  Need for regional and North American collaboration  Effectiveness of response coordination strategies |
| **4** | Communicable disease control | Labour issues |
| **5** | Communication | Public health interface with primary care  Social media  IT issues and solutions in health |
| **6** | Education, training and exercises | ICS/IMS implementation  Decontamination exercises  Foresight planning  Flexibility  Emergency decision-making for public health practitioners  Logistics issues and challenges |
| **7** | Public health considerations for sheltering and evacuation | Feasibility of rapid mass evacuation in a Canadian city  Public preparedness requires extensive education programming; skillsets found in other disciplines  Functional needs assessments |
| **8** | Quality improvement and performance standards | Psychosocial supports for first responders and receivers |
| **9** | Surveillance, epidemiology and public health information | Interface of public health with primary and emergency care  Foresight planning  Electronic health records  Psychosocial impacts  Resilience |
| **10** | Vulnerable populations | Ethics considerations  Specific populations  Utility of vulnerability assessments  Engagement with community-based organizations |
